# An Oncological Emergency: Severe Type B Lactic Acidosis From Warburg Effect in Diffuse Large B-cell Lymphoma

**DOI:** 10.7759/cureus.26557

**Published:** 2022-07-04

**Authors:** Raghavendra Sanivarapu, Pratap Kumar Upadrista, Jonathan Otero-Colon, Kunal Shah, Bair Cadet, Qi Tao, Javed Iqbal

**Affiliations:** 1 Department of Pulmonary and Critical Care Medicine, Nassau University Medical Center, East Meadow, USA; 2 Department of Internal Medicine, Nassau University Medical Center, East Meadow, USA; 3 Department of Pathology, Nassau University Medical Center, East Meadow, USA; 4 Department of Nephrology, Nassau University Medical Center, East Meadow, USA

**Keywords:** oncological emergency, warburg effect, dlbcl, type b lactic acidosis, lactic acidosis

## Abstract

Lactic acidosis is the most common anion gap metabolic acidosis in critically ill patients. Type B lactic acidosis is most commonly seen with hematological malignancies, especially lymphomas. It is considered an oncological emergency and is associated with high mortality and poor outcomes if not treated promptly. Here, we present the case of a 48-year-old male who developed Type B lactic acidosis secondary to newly diagnosed diffuse large B-cell lymphoma. This case highlights the importance of including Type B lactic acidosis in the differential diagnosis in a patient with unexplained lactic acidosis and hypoglycemia with otherwise vague symptoms and the need for a thorough search for quick diagnosis and early management.

## Introduction

Lactic acidosis is the most common anion gap metabolic acidosis in critically ill patients [1]. It occurs when the production of lactic acid exceeds clearance [2]. Most of the lactate (80-90%) is cleared by the liver through gluconeogenesis while the remaining is cleared by the kidneys [3,4].

Type B lactic acidosis is most commonly seen with hematological malignancies, especially lymphomas. It is considered an oncological emergency and is associated with high mortality and poor outcomes if not treated promptly [2].

Here, we present the case of a 48-year-old male who developed Type B lactic acidosis from newly diagnosed diffuse large B-cell lymphoma (DLBCL).

## Case presentation

A 48-year-old Hispanic man presented with a three-day history of left-sided headache, left jaw pain and difficulty speaking, and tongue deviation to left. He sustained a fall six months back without loss of consciousness but noticed some weakness in his right leg and tongue, which resolved in a few hours, and did not seek medical attention. He endorsed about 30 lbs weight loss within a six-month period.

On admission, the patient had a blood pressure of 118/76 mmHg, a pulse rate of 98 beats/minute, respiratory rate of breaths 14/minute, and oxygen saturation of 98% on room air and was afebrile. Physical examination was significant for palpable non-tender supraclavicular, axillary, posterior cervical, and bilateral inguinal lymphadenopathy and decreased breath sounds at the left lung base associated with a dull note on percussion. A detailed neurological examination revealed decreased left eye abduction and decreased light reflex in the left eye and deviation of the tongue to the left. The remainder of the examination was unremarkable.

**Figure 1:**
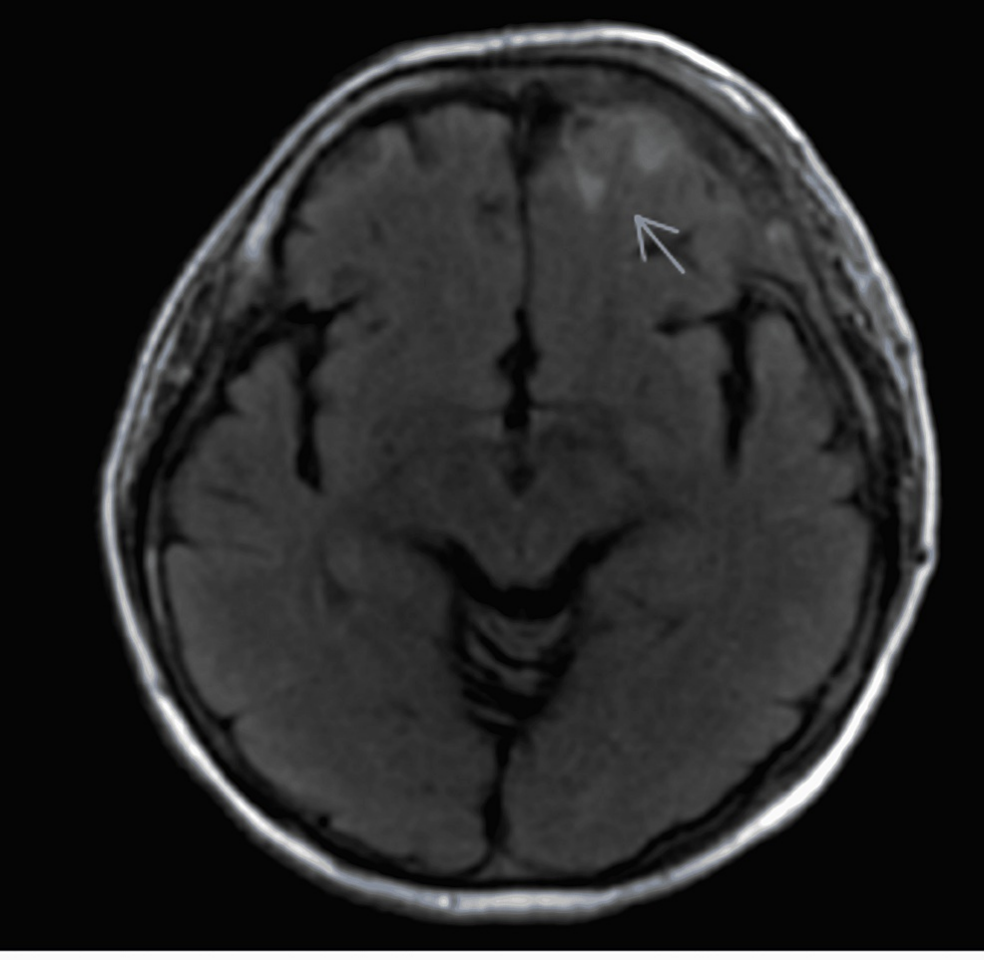
MRI showing a small hyperintense lesion in the left frontal cortical region. MRI: magnetic resonance imaging

**Figure 2:**
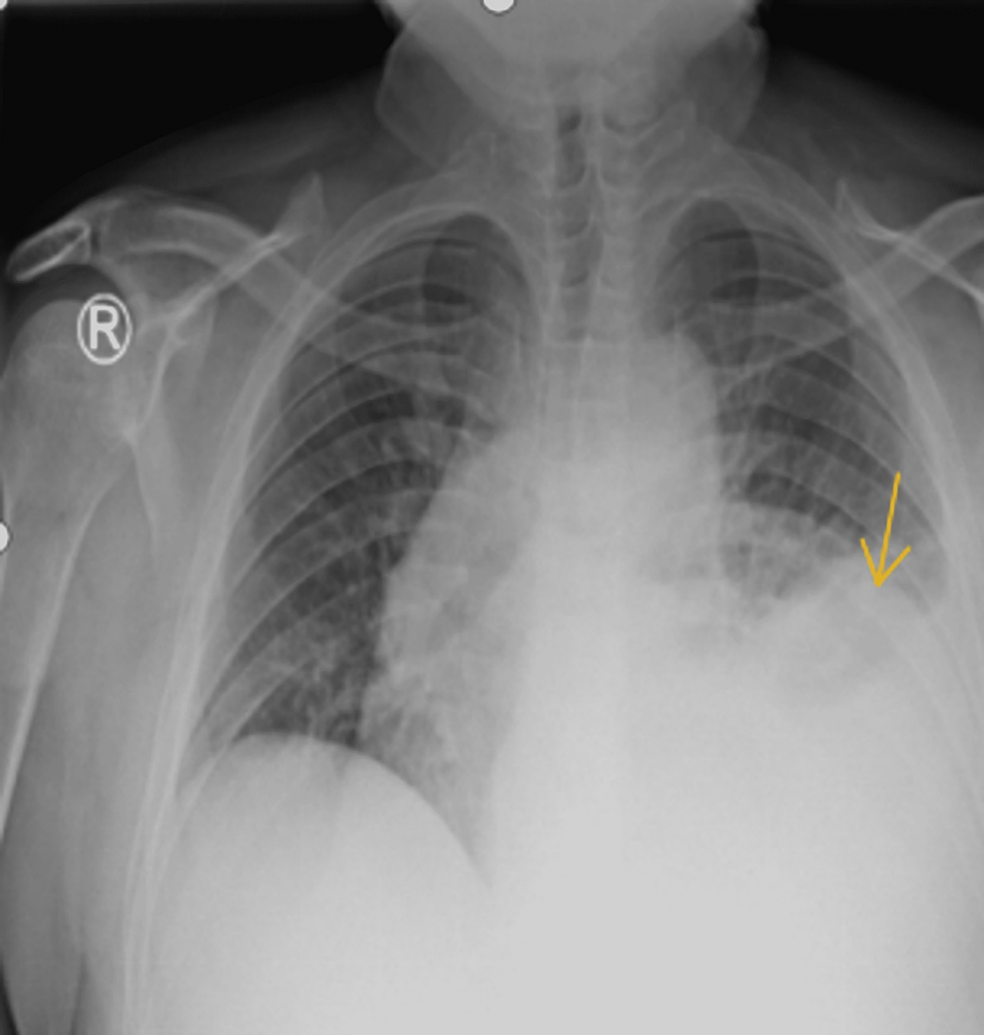
Chest X-ray showing left pleural effusion.

An initial computed tomography (CT) of the head demonstrated a sub-acute on chronic left subdural hematoma associated with a left frontal contusion. Magnetic resonance imaging (MRI) of the brain with angiography demonstrated a multifocal patchy enhancing marrow replacing the process of the calvarium representing osseous metastatic disease and associated soft tissue extension beyond the osseous margins (Figure 1). Pachymeningeal thickening and enhancement at the left frontal convexity; and a small T2/fluid-attenuated inversion recovery (FLAIR) signal abnormality at the left frontal lobe cortical region indicated potential sequelae of parenchymal extension of disease. The presence of generalized lymphadenopathy and radiological evidence of a probable metastatic disease prompted a thorough evaluation. His chest X-ray showed hilar fullness with left-sided pleural effusion, as shown in Figure 2.

**Figure 3:**
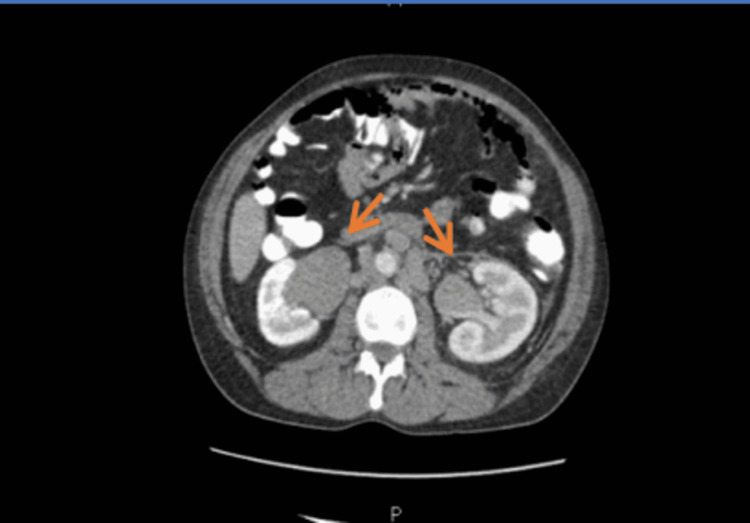
CT abdomen showing bilateral lymphadenopathy with hydroureteronephrosis (arrows). CT: computed tomography

CT thorax, abdomen, and pelvis with contrast showed a large left pleural effusion with pleural thickening, mediastinal, bilateral hilar, axillary, cervical, and abdominal lymphadenopathy along with enlarged spleen with innumerable hypoattenuating lesions and bilateral moderate hydronephrosis due to compression of ureters from lymph nodes (Figure 3).

His initial blood work revealed microcytic anemia (hemoglobin = 8.7 g/dL), thrombocytosis (495 K/mm^3^), elevated erythrocyte sedimentation rate (ESR) (78 mm/hour), and lactate dehydrogenase (LDH) levels (471 U/L), acute kidney injury (AKI) with a serum creatinine of 1.9 mg/dL, and mild transaminitis. Peripheral smear was suggestive of anisocytosis, poikilocytosis, polychromasia, ovalocytes, burr cells, schistocytes, target cells, and teardrop cells.

Given the patient’s large left pleural effusion, 1,500 mL of dark yellow-colored fluid was drained. The pleural fluid analysis showed an exudative effusion with lymphocyte predominance (70%) with 3% atypical cells.

**Figure 4:**
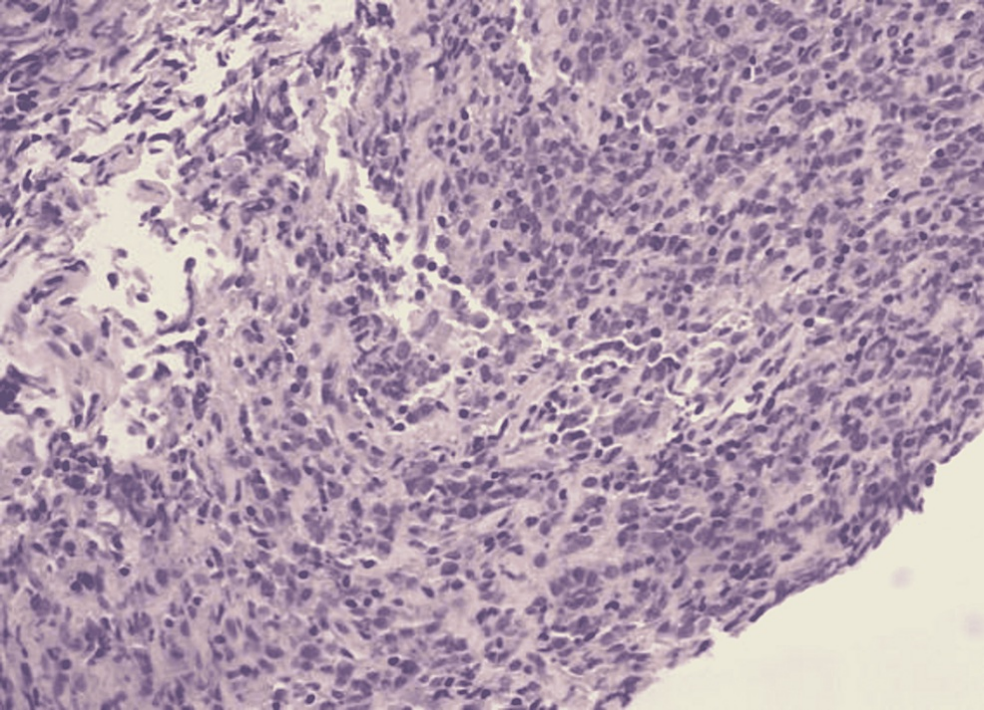
Lymph node core biopsy showing lymphoid tissue with mixed small and intermediate-sized lymphocytes.

**Figure 5:**
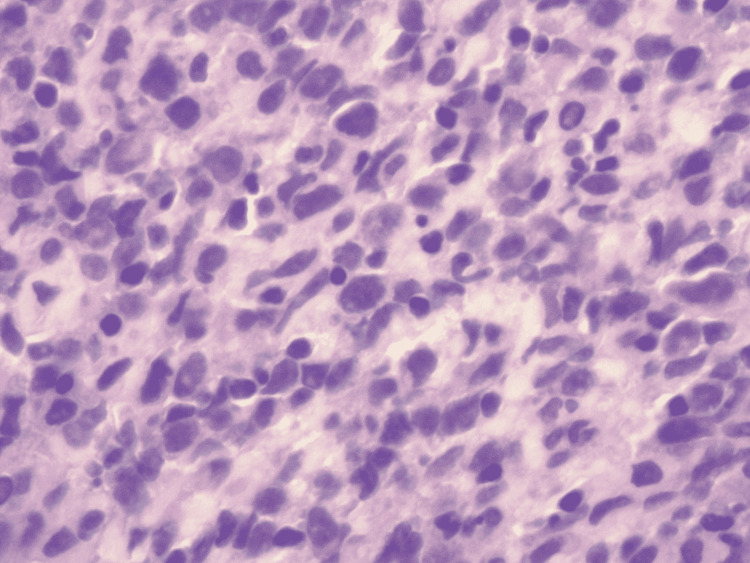
Atypical large lymphocytes showing nucleomegaly with inconspicuous nucleoli.

The patient underwent a left axillary lymph node biopsy, bone scan, and serum protein electrophoresis (SPEP) for malignancy workup. A bone scan showed marrow infiltration at multiple levels; however, no overt lesions were noticed. SPEP had a normal pattern and no monoclonal pattern was seen. Left axillary lymph node biopsy revealed a low-level atypical B-cell population with immunohistochemistry positive for CD20, CD79a, PAX5, and sBCL2 dim and bright consistent with DLBCL (Figures 4, 5).

The patient had an extensive hospital course complicated with hypercalcemia and worsening transaminitis and recurrent left-sided malignant pleural effusion. He also developed high anion gap metabolic acidosis secondary to lactic acidosis and hypoglycemia despite adequate oral intake. Workup for viral hepatitis was negative and abdominal ultrasonogram showed no abnormalities.

**Figure 6:**
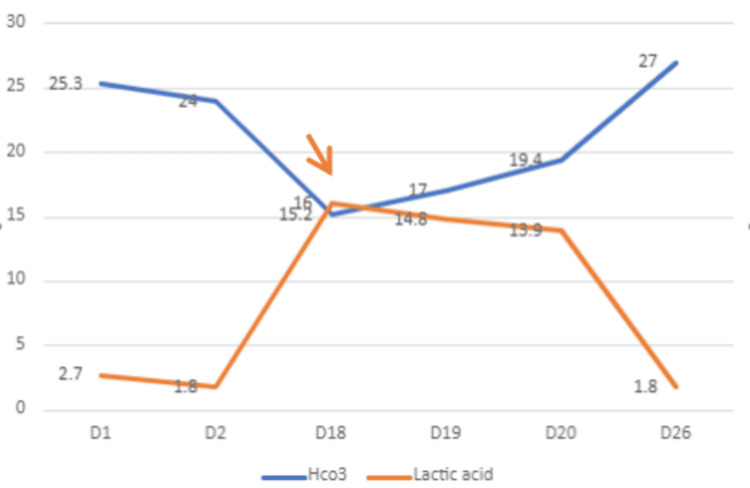
The trend of lactic acid and HCO3 levels over the course of hospital stay with an arrow indicating initiation of hemodialysis.

His lactic acidosis worsened with a peak level of 16, as depicted in Figure 6. He was hemodynamically stable with a blood pressure of 112/68 mmHg, a heart rate of 98 beats/minute, and his oxygen saturation was 98% on room air despite high lactate and severe acidosis. His blood glucose levels were noted to be low at 38 mg/dL. He was started on intravenous (IV) fluids and one dose of zoledronic acid for hypercalcemia. He was also started on an IV sodium bicarbonate drip and a high dose of IV thiamine. He was started on emergent hemodialysis (HD) along with chemotherapy with an R-CHOP regimen (rituximab, cyclophosphamide, hydroxydaunorubicin hydrochloride (doxorubicin hydrochloride), vincristine (Oncovin), and prednisone), which led to improvement in the patient’s Type B lactic acidosis over the course of a few days.

## Discussion

Luft et al. defined lactic acidosis as lactate levels of more than 5 mmol/L associated with metabolic acidosis with a pH of less than 7.35 [5]. There are two types of lactic acidosis based on pathophysiology: Type A, which is the most common type and is due to poor tissue oxygenation and hypoperfusion, and Type B, which is seen in conditions with normal oxygenation and perfusion and is usually a result of drugs or toxin that interfere with normal cellular metabolism or due to nutritional deficiency [6-8].

Type B lactic acidosis is a dreaded complication associated with solid (15%) and hematological malignancies (85%) [9]. A retrospective study by Friedenberg et al. showed that type B lactic acidosis is most commonly associated with lymphomas, multiple myeloma, and leukemia and is associated with high mortality (>90%) and has a very poor prognosis [10,11].

The underlying mechanism for type B lactic acidosis in hematological malignancies can be multifactorial but can be explained by the “Warburg” effect also termed aerobic glycolysis first described by Otto Warburg. The mechanism involves tumor cells having very high glucose uptake compared to non-cancerous cells, yet preferentially producing lactate via anaerobic metabolism despite normoxic and normotensive conditions [12].

Our patient initially presented with subdural hematoma, significant weight loss, and generalized lymphadenopathy. His laboratory results were significant for microcytic anemia, elevated LDH levels, and transaminitis. Acute kidney injury is presumed secondary to obstructive uropathy caused by adenopathy and radiological evidence of a large pleural effusion, metastatic disease in the marrow and intracranial metastatic lesion, and generalized lymphadenopathy and splenomegaly. Initial differential diagnoses included lymphoma, leukemia, and multiple myeloma. Biopsy of lymph node clinched the diagnosis of DLBCL. He did not have any hypoxia and was hemodynamically stable when he developed lactic acidosis, warranting us to think in terms of type B lactic acidosis. A search for infectious etiology was ruled as blood cultures were negative.

A brief review by Claudino et al. in a case report suggested the likely cause for hypoglycemia is a direct result of excess consumption of glucose by tumor cells [13]. However, the treatment should not be focused on supplementing excess glucose as it could worsen the lactic acidosis from over-consumption by tumor cells, a phenomenon described as hyper-Warburgism [14].

As a high tumor burden is causing lactic acidosis, the mainstay of treatment is cytoreductive chemotherapy, as demonstrated by Chan et al. [15] in their retrospective literature review of patients with lymphoma, who developed Type B lactic acidosis and by Silos et al. [11], in their review of literature of patients with hematological malignancies, who developed Type B lactic acidosis. However, it did not cause resolution in all the cases, as reported by Kestler et al. [16]. Ruiz et al. suggest that treatment modalities such as hemodialysis, thiamine, and sodium bicarbonate infusion should be considered while a response to chemotherapy ensues but their efficacy is controversial [9]. Our patient received three cycles of hemodialysis, thiamine, and sodium bicarbonate infusion, while he was started on chemotherapy, following which the patient’s lactic acidosis improved.

## Conclusions

Type B lactic acidosis is an oncological emergency that warrants prompt diagnosis and aggressive treatment due to high mortality and poor prognosis. Currently, the most common treatment strategy is effective cytoreductive chemotherapy to reduce the high tumor burden that precipitated lactic acidosis. However, even with aggressive chemotherapy, mortality rates are high. Hence, unexplained lactic acidosis and hypoglycemia with otherwise vague symptoms should prompt a thorough search for quick diagnosis and early management.
